# Operating Room Usage Time Estimation with Machine Learning Models

**DOI:** 10.3390/healthcare10081518

**Published:** 2022-08-12

**Authors:** Justin Chu, Chung-Ho Hsieh, Yi-Nuo Shih, Chia-Chun Wu, Anandakumar Singaravelan, Lun-Ping Hung, Jia-Lien Hsu

**Affiliations:** 1 Department of Computer Science and Information Engineering, Fu Jen Catholic University, New Taipei City 242062, Taiwan; 2 Department of General Surgery, Shin Kong Wu Ho Su Memorial Hospital, Taipei 111045, Taiwan; 3 Graduate Institute of Applied Science and Engineering, Fu Jen Catholic University, New Taipei City 242062, Taiwan; 4 National Taipei University of Nursing and Health Sciences, Taipei City 112, Taiwan

**Keywords:** operating room usage time, scheduling, machine learning, XGBoost

## Abstract

Effectively handling the limited number of surgery operating rooms equipped with expensive equipment is a challenging task for hospital management such as reducing the case-time duration and reducing idle time. Improving the efficiency of operating room usage via reducing the idle time with better scheduling would rely on accurate estimation of surgery duration. Our model can achieve a good prediction result on surgery duration with a dozen of features. We have found the result of our best performing department-specific XGBoost model with the values 31.6 min, 18.71 min, 0.71, 28% and 27% for the metrics of root-mean-square error (RMSE), mean absolute error (MAE), coefficient of determination ($R^{2}$), mean absolute percentage error (MAPE) and proportion of estimated result within 10% variation, respectively. We have presented each department-specific result with our estimated results between 5 and 10 min deviation would be more informative to the users in the real application. Our study shows comparable performance with previous studies, and the machine learning methods use fewer features that are better suited for universal usability.

## 1. Introduction

### 1.1. Background

Surgery operation rooms (ORs) are considered valuable in terms of high medical and human resource cost. Although the OR is a limited and high-cost resource, it also earns higher profits for hospitals [1]. A previous study tries to find the direct and indirect costs of 1 minute of OR time and also analyzes the cost of facility features, direct, and indirect costs related to the OR. Based on the California hospital financial statements, the OR time is measured at $36 to $37 per minute [2]. This signifies that the efficient use of the operation room is an important research topic. The booking and scheduling of an operating room can be very complicated due to the need for collaboration between different departments. An optimized OR room schedule does not rely only on the available operation room but also the other post-op resource’s availability [3]. In order to increase OR room usage efficiency, various research studies have been conducted from several perspectives such as improving the OR scheduling [4], identifying causes of delay [5], optimizing workflow and standardizing equipment [6]. In this study, we focus on the difficulties experienced while improving efficiency through the scheduling process. Many hospitals have their own policy to optimize their OR scheduling, however, the effectiveness of the policy rely on accurately estimated usage time of OR rooms. In general, the OR room usage time heavily depends on the doctor’s personal experience. In some cases, there are certain hospitals that still follow the queued method where every patient who needs surgery is prioritized in order and waits until their turn. We believe that a precise estimation of operation (OP) time can help both medical institutions and patients. The accurate estimation of operation time can potentially improve patients quality of healthcare by reducing their wait time and proper reschedule.

### 1.2. Previous Studies

Surgical procedures consist of pre-surgery, surgery, and post-surgery. Pre-surgery is the process of setting up and preparing for the operation. The duration of surgery includes the anesthesia procedure and surgical procedure. Pre-operative and post-operative times are included in the non-operative period, whereas the operative phase comprises the total operation time [7,8,9]. Patients are categorized into inpatients, outpatients and emergency cases. Surgery is scheduled in advance for both inpatients and outpatients based on the multiple resource availability. Advance scheduling is not possible for emergency patients. Anesthetists, nurses, surgeon groups, medical technicians, medical supplies, and operating rooms (ORs) are involved in a surgical procedure [10]. The OR scheduling process considers time-based constraints to measure performance [11]. Excess time and idle time are considered essential metrics in a few research studies. The precise estimation of the operating room usage time can be helpful to the patients. Surgical durations are difficult to predict in advance, which may cause long OR overtime and idle times. OR overtime may cause surgery cancellation and failure of the healthcare experience. Idle time leads to poor usage of OR capacity [12,13,14]. Multiple studies have attempted to implement new prediction models for estimating operation room usage time in past decades. Recently, the coronavirus disease 2019 (COVID-19) caused a high number of cancellations of surgical operations which increased the wait lists and in turn affected the patient healthcare experience. During this pandemic situation, recent inspections have used surgeon-specific data to enhance surgery bookings using machine learning model [15]. With the intention of lowering surgical costs, a modular artificial neural network-based solution is created to anticipate surgical duration using the procedure and medication data [16]. Another neural network-based approach independently balances the data and also utilizes the MLP model to provide duration prediction systems for surgery and anesthesia [17]. Eijkman et al. have created a model with linear mix modeling on over 17 thousand entries of data consisting of different features including patients’ personal psychological information [18]. Their proposed model is a simple linear regression and achieved good improvement in the estimation of operative time compared with previous studies. Similarly, Edelman et al. incorporated the linear regression models on over 79 thousand records of OR room usage data with fewer features but these results heavily rely on anesthesia-related characteristics [19]. Bartek et al. developed models to forecast case-time duration with the help of linear regression and supervised machine learning [20]. Their model took advantage of three different data groups named all-inclusive, service-specific, and surgeon-specific to predict case-time duration. The XGBoost model has scored better results and outperformed the traditional linear regression models in every category. The model needs detailed patient information which leads to backlogs when there is no data related to patient physiological information. Past literature utilizes computer simulation to determine OR scheduling [4]. Their study comprises the wait time and surgery time as essential parameters. Block time is decided based upon the expected total duration of inpatient and outpatient cases which helps to enhance OR utilization by adopting OR scheduling tricks. A latest study considers demographic data, operation procedures, anesthesia type and the level of expertise of the primary surgeon as suitable features for the prediction of length of surgery [21]. Bandi et al. has handled a multifaceted problem that includes OR staffing and scheduling which paves the way to create new criteria for constructing online algorithms termed “the robust competitive ratio” [22]. Li et al. handled the similar OR schedule re-optimization problem in their approach which significantly improves an existing method by addressing the surgeon’s preferences as well as all of the limitations [23]. A recent research proposes an OR scheduling framework based on patients’ preferences. This investigation considers certain attributes such as professional experiences, same ethnicity, same gender, communication skills and several criteria have been involved to produce this framework [24].

### 1.3. Aim of This Study

This study applied machine learning and deep learning methods to the surgery room usage records to explore various options for improving OR room usage efficiency. Our work aims to construct prediction models to accurately predict the OR room usage time and compare the performance of different models. All the models are restricted to using a few simple parameters that are available before the surgery. Due to the lacking of detailed personal information records such as age, gender and medical history, the model will not include any patient’s personal information. The nature of this study in which restricted parameters are involved to reveal our method can be implemented in most cases.

## 2. Materials and Methods

### 2.1. Data Source

This study makes use of surgery room usage records from Shin Kong Wu Huo-Shih Memorial Hospital from January 2015 to September 2019. The raw data collection contains a total of 124,528 entries of room usage records, including 112 columns of various parameters. There are no patient information and identifiable information in the data collection. The table comprises department numbers, involved personnel (including doctors and nurses), patient status, surgery urgency, discovery and doctor’s comments during the procedure, timestamps, and procedure IDs. Appendix A contains a full structure of the raw data table.

### 2.2. Data Preprocessing

Deletion and interpolation are the two primary processing methods for handling missing data problem [25]. The deletion process simply removes missing data and retains the remaining dataset for further analysis. This is a simple and feasible solution to deal with the missing data. The impact of deleting missing data is less when the amount of missing data is small [26]. In total, we exclude columns of missing values, ID-like parameters, timestamp parameters and comments during the data preprocessing. Multiple types of data are not suitable for applying interpolation techniques to recover the raw data. We choose the deletion method to handle the missing data issue. Many of the 112 columns are eliminated due to being empty or in an unsuitable format such as date format or timestamp format. Features that are not available prior to the procedure are eliminated to avoid confounding the response. Finally, all ID-like parameters such as doctor and nurse ID are also excluded, thus limiting the number of features utilized in this study. A few additional characteristics are created, resulting in a total of 15 features used in this study. A total of 62 columns were eliminated due to being empty, and a total of 13 columns of timestamps are also removed. A total of 17 columns indicating personnel IDs, hardware IDs, and room numbers are removed too. A total of ten columns of text-based comments and diagnoses are removed as well. There are a total of 5 new feature columns generated. The prediction target of total surgery room usage time is calculated based on the time stamp difference between entering and exiting the room. The number of involved doctors and nurses is divided into two columns to replace individual IDs. The total number of the procedure is also calculated. Moreover, the number of days since the patient had their last surgery is calculated.

The resulted 15 features columns are as follows:

- *ODR_DEPT*: Department’s ID;
- *ODR_WOUD*: Patient’s wound cleanness;
- *ODR_ASA*: Patient’s anesthesia risk;
- *num_M_DR*: Total number of doctors will be partaking the surgery;
- *num_DN&WN*: Total number of nurses (scrub nurse and circulation nurse) will be partaking in the surgery;
- *num_OP*: Total number of planned procedures;
- *Day_since_last*: Number of days since patient’s last surgery;
- *ODR_PAYK_cat*: Patient payment category (Health insurance status);
- *ODR_PSRC_cat*: Patient source category (Inpatient, Outpatient, Emergency room );
- *ODR_EFLG_cat*: Category (if it is emergency surgery);
- *ODR_OP_1_cat*: Procedures id of the first procedure to be performed;
- *ODR_OP_2_cat*: Procedures id of the second procedure to be performed;
- *ODR_OP_3_cat*: Procedures id of the third procedure to be performed;
- *ODR_OP_4_cat*: Procedures id of the forth procedure to be performed;
- *ODR_Total_time*: Total time (in minutes) spent in OR room.

A total of 987 entries of data are excluded from the study for having negative time spent in the OR room. Another 65 entries of data are excluded from the data for being an extreme outlier with more than 800 minutes of surgery time, thus leaving a total of 123,476 records. Label encoding methods are then applied to transform the categorical data into a usable format for training the models. The data entries are further sorted according to their department numbers to construct a department-specific data set.

**Figure 1:**
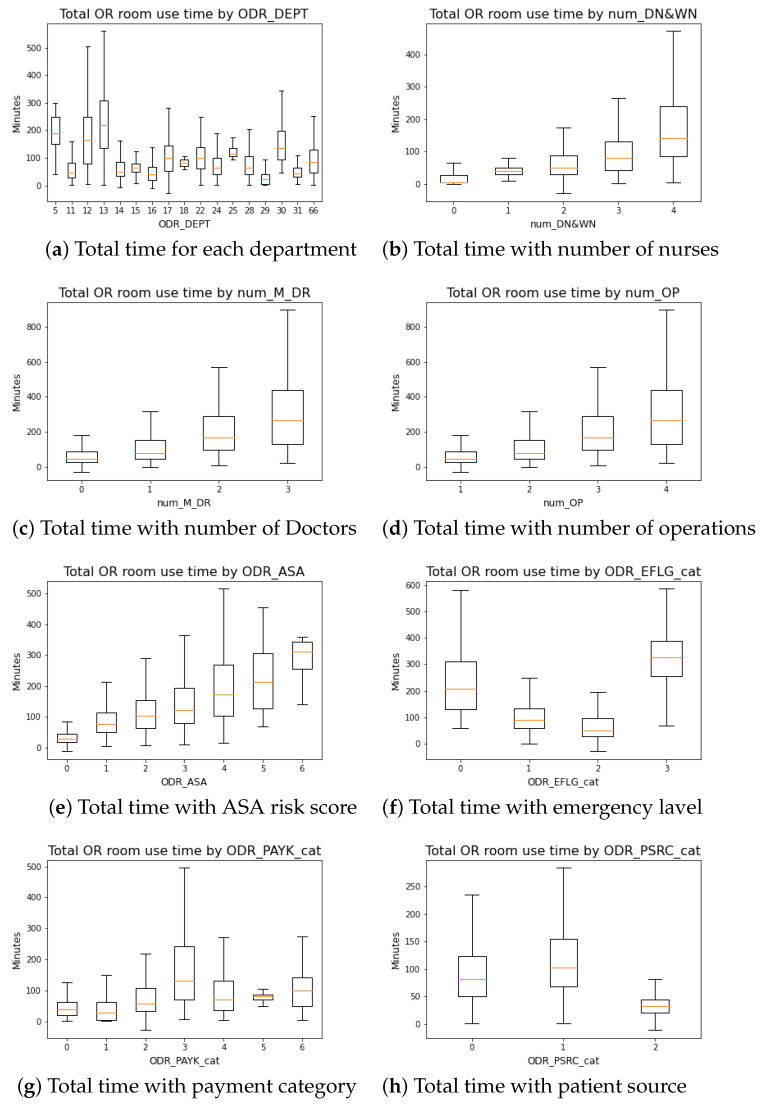
Feature demographic.

Table 1 summarizes the overall data characteristic profile when grouped by different departments, while Figure 1 highlights each feature’s correlation with total OR room usage time distribution in form of a box-plot. Figure 1 illustrates that the total time is strongly correlated with the number of participating doctors, nurses, and operations scheduled. The anesthesia risk level has a high association with total usage time. Each different department also has its unique overall distribution of total time spent in the OR room.

### 2.3. Methods

**Figure 2:**
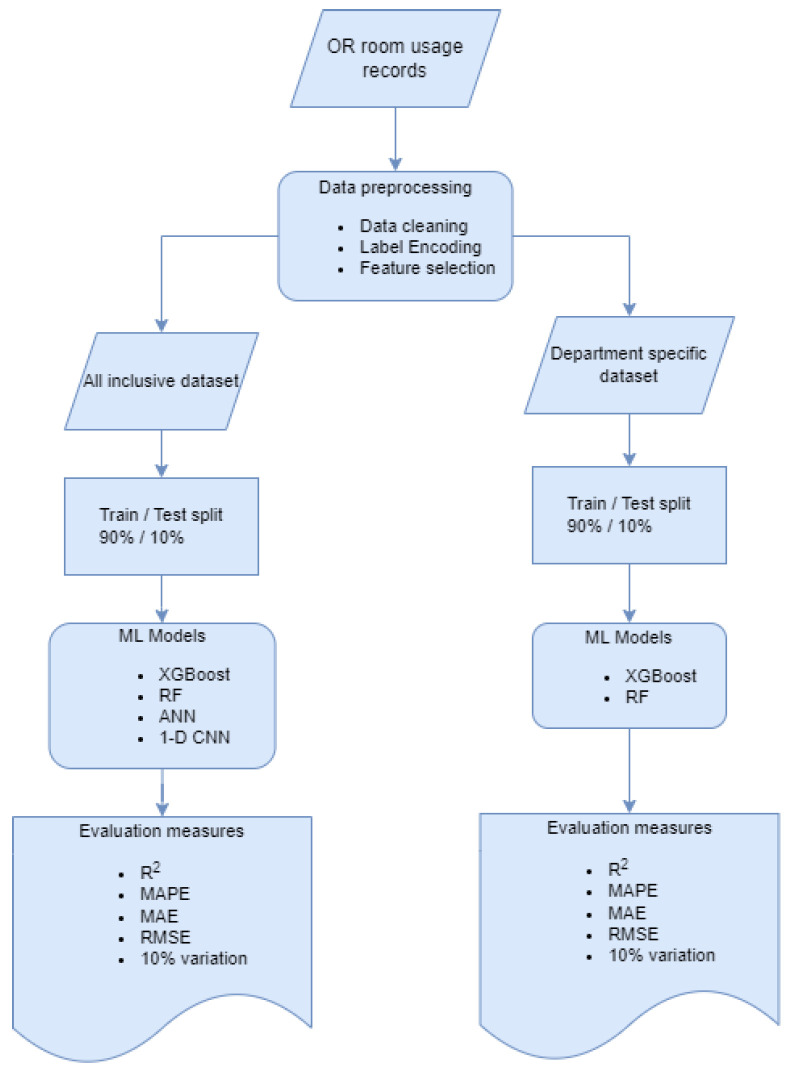
Process flowchart of data and model.

In this study, we apply four machine learning models including XGBoost (Extreme Gradient Boosting), Random Forest, Artificial Neural Network (ANN), and 1-dimensional Convolution neural network (1dCNN) to forecast OR usage time. The first two methods are based on decision trees, while the last two methods are based on deep neural networks. Initially, we prepare an all-inclusive dataset that contains all data records to optimize a more generic model. The all-inclusive model is used to do a preliminary evaluation of the model’s performance. The second department-specific dataset trains a dedicated model for each department separately. The departments with a thousand records are used to ensure each department’s model has sufficient learning data for the department-specific models. Having two different datasets reveal whether the model benefits from cross-department data. The step-by-step process of this study is depicted in Figure 2. The flow chart shows the workflow of this study from raw data to final performance measurement.

Tianqi Chen originally releases XGBoost in 2014, and it has since become a popular open-source package used by data scientists. XGBoost is a scalable end-to-end tree boosting machine learning system that utilizes fewer resources than existing systems. The tree boosting method builds a series of trees that improves based on their predecessor’s prediction error. Iterative training is applied to improve on prior iterations by lowering errors. Since 2015, XGBoost models have won multiple machine learning challenges, which proves their performance and efficacy [27]. We utilize the grid search approach to obtain the optimal parameters for each decision-tree-based model in this study.

Random Forest is a decision tree-based machine learning model that aggregates numerous findings using ensemble learning. The Random Forest model uses the bagging approach, which creates a vast number of randomly generated individual decision trees and uses a majority vote to determine the prediction result. Each of the randomly generated trees is generated equally with a subset of the training data. Unlike a boosting tree, bagging does not have a sequential relationship between the trees. The goal is to use slightly different sets of data and create a vast number of distinct trees hoping that they all agree on the same outcomes. The model is known for being resistant to noise, outliers, and over-fitting. The grid search approach is applied to select the optimal parameter for each model, as previously mentioned [28].

An artificial neural network (ANN) follows a neural network structure that replicates human brain functions by connecting various neurons. ANN is one of the fundamental methods of Artificial Intelligence, consisting of layers of varying numbers of neurons that are completely linked between layers (each neuron is connected to every neuron on the following layer).

Each neuron takes inputs and processes with corresponding weights and then passes them through an activation function to generate their output. The weights are adjusted based on the error value through the backpropagation, which happens continuously until the divergence.

**Figure 3:**
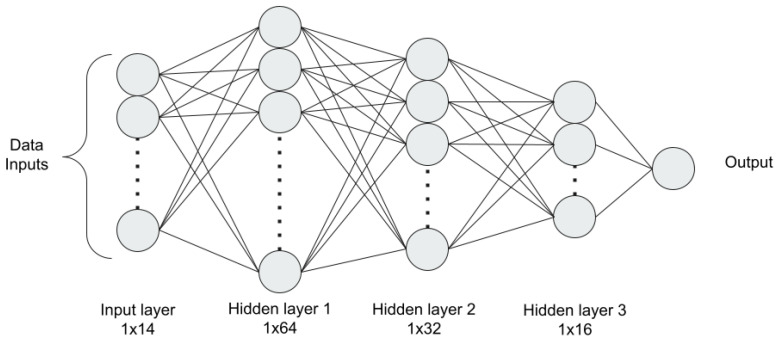
ANN model structure.

The ANN architecture used in this study is depicted in Figure 3 [29]. The model includes an input layer, three hidden layers, and an output layer. The input layer accepts a dimension of 1 × 14 as input. The three hidden layers each consist of 64, 32, and 16 neurons. For the hidden layers, rectified linear units (ReLU) are used as their activation functions. Batch normalization is applied to the output of each hidden layer before feeding them into the next layer. The output layer with one single neuron takes the output from the last hidden layer and used a linear activation function to return a prediction result. The model uses mean squared error as its loss function with adam optimizer.

**Figure 4:**
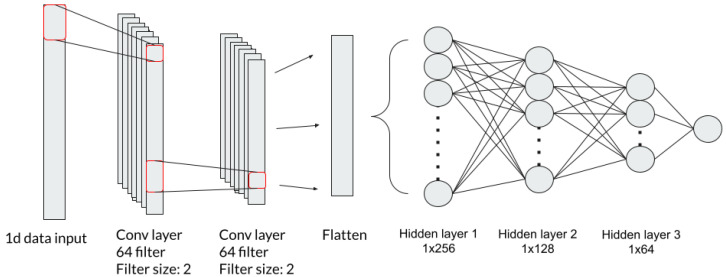
1dCNN model structure (Visual representation of the model architecture with two one-dimensional CNN layers followed by three fully connected layers used in this study).

A convolutional neural network (CNN) is a widely used deep learning model structure that consists of numerous layers of convolutional and sub-sampling layers followed by the aforementioned hidden layers of ANN. The convolutional layers are capable of extracting features with their filters from the input data. The traditional CNN is often used for solving image-related problems whereas 1-dimensional CNN is a subset of convolutional neural networks, which takes one-dimensional inputs instead of two-dimensional images. 1dCNN architecture is presented in Figure 4 [30]. The input layer accepts a dimension of 1 × 14 as input. A total of 2 pairs of one-dimensional convolution layers are placed in the architecture. Each convolution layer has a filter size of 64 and a kernel size of 2. Batch normalization is applied after each convolution layer and then passed through a ReLU activation function before entering into the next layer. The high-dimensional features from the convolution layers are flattened before being entered into three hidden layers of ANN. The hidden layer consists of 256, 128, and 64 neurons and also employ ReLU as their activation function. Batch normalization is applied followed by the output of each hidden layer. At the end, the output layer with a single neuron takes the output from the last hidden layer and is processed with a linear activation function to produce prediction result.

All models are constructed based on 9:1 training and testing set splits. The decision tree model also uses 5-fold cross-validation on the 90% of its training set and is evaluated based on the remaining 10% testing set. The neural-network-based models are trained and tested based on the 90% training set and 10% testing set with different random seeds 5 times and evaluated based on the best model result.

For a purpose of comparison with previous studies, the model performances are evaluated in the form of mean-absolute error (MAE), mean-absolute-percentage error (MAPE), root-mean-squared error (RMSE), coefficient of determination ($R^{2}$), percentage within 10% variation (±10%), and percentage of the samples within 5 or 10 min (±5 min, ±10 min).

## 3. Results

Initially, we compare the performance of each method among our all-inclusive models to reveal the performance of each ML model. We show both the individual model performance of each department and the weighted sum of all the ML approach models to show the performance of the department-specific model. In addition, we provide the comparison table with previous studies for a more clear understanding.

### 3.1. All-Inclusive Model Results

**Figure 5:**
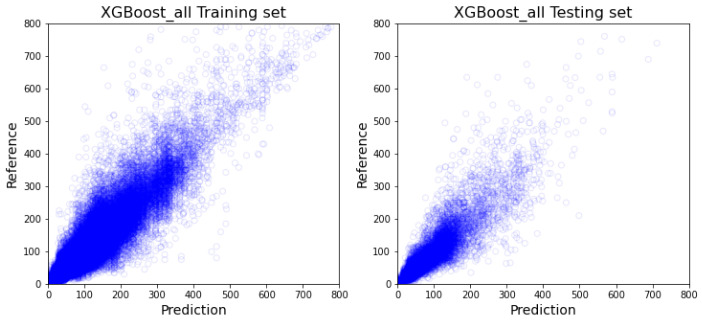
All-inclusive model with XGBoost (The visualization of training and testing data for all-inclusive model with XGBoost).

**Figure 6:**
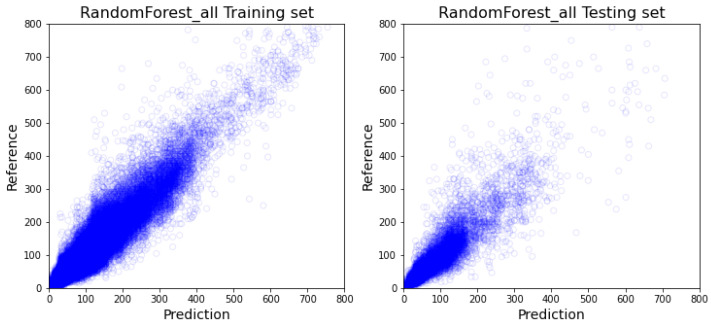
All-inclusive model with Random Forest (The visualization of training and testing data for all-inclusive model with random forest).

**Figure 7:**
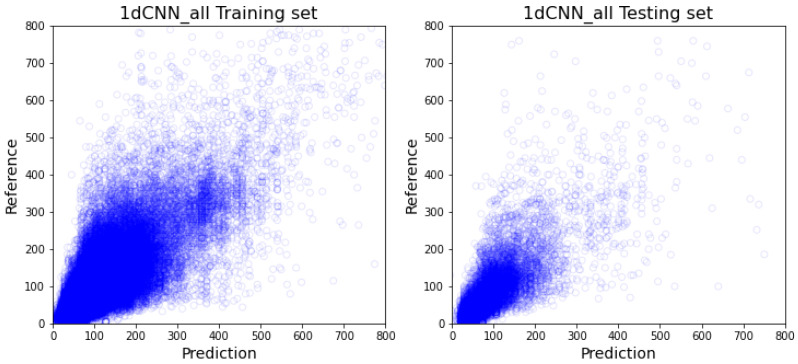
All-inclusive model with 1dCNN (The visualization of training and testing data for all-inclusive model with 1dCNN).

**Figure 8:**
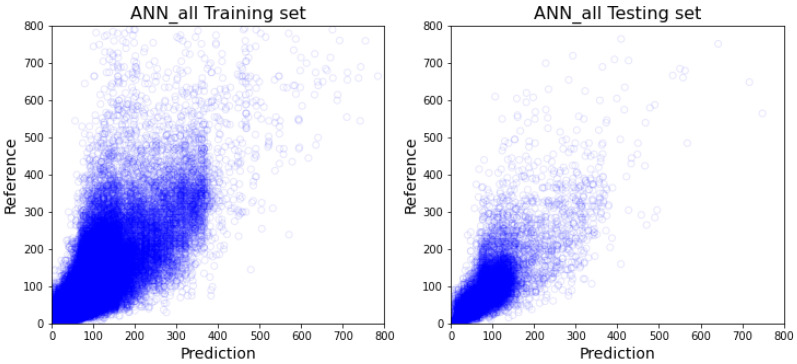
All-inclusive model with ANN (The visualization of training and testing data for all-inclusive model with ANN).

As demonstrated in Table 2, out of the all-inclusive models, the XGboost model (Figure 5) and random forest model (Figure 6) have similar performance on every comparable score, and more specifically outperform 1dCNN (Figure 7) and ANN (Figure 8) models. The $R^{2}$ value of XGBoost and the random forest is approximately 0.1 higher than 1dCNN. 1dCNN has around 10% fewer forecasted data falls within 10% variance. These results suggest that based on our data, deep-learning ANN and 1dCNN models may not be the best ML models for accurately predicting OR room usage time. The two approaches were not further studied using department-specific models due to the poor performance.

### 3.2. Department-Specific Model Results

Only 11 departments out of 17 are utilized to build the department-specific models, each containing over a thousand records of OR room utilization data. A dedicated model is constructed with each model for each department. Random Forest and XGBoost based department-specific model results are described in Table 3. The tables include detailed model performance for each department with a weighted average (denoted with (weighted)). The scatter plot visualizes the detailed training and testing performance of every individual department’s model of XGBoost and Random Forest, which can be demonstrated in Appendix A.

**Figure 9:**
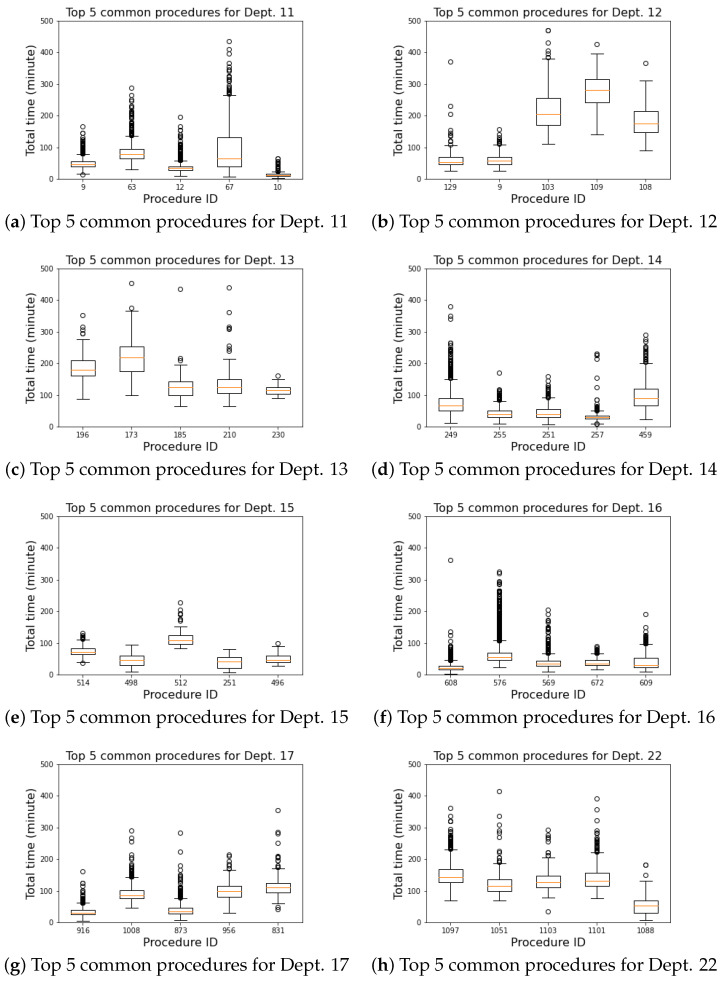

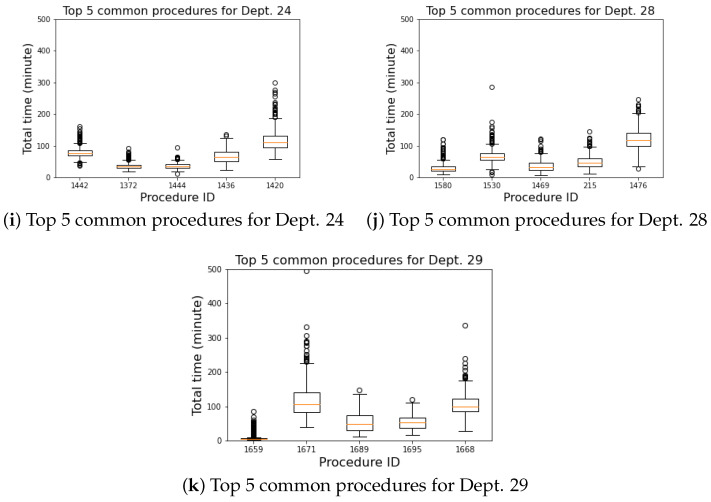
Total time distribution of the top 5 commonly performed procedures for each department.

The results illustrate the significant performance difference between departments. The large difference between departments shows the single all-inclusive model is not a best way to handle all the cases. Figure 9 shows the box plots of their top five commonly performed procedures to explore the difference in detail. The box plots demonstrate the variation of common procedures belonging to the same department. The variation of these plots delivers the idea behind the challenges of the department-specific model.

## 4. Discussion

### 4.1. Comparative Study

Table 4 illustrates the results of prior research and our method with mutually available parameters for comparison.

In terms of MAPE, our department-specific model and Bartek et al.’s [20] service-specific model seems similar; however, our model has a relatively low percentage of samples according to ±10% and $R^{2}$ value. These three evaluation metrics are insufficient to judge a model which outperforms based upon the percentage of samples within ±10% which is heavily influenced by data distribution. We have discovered that evaluating a model’s performance by its ±10% variation is not optimal, especially on short time procedures. A shorter procedure can surpass the 10% range by having any minor delay far easier than a long procedure. The time cost of any extra human action or deviation does not scale up or down based on the duration of the operation. For instance, department 13 has a poor RMSE, MAE, and $R^{2}$ score but achieves a high percentage of predicted results within ±10% variance. Without knowing the data distribution, it is hard to compare previous research models with an objective view.

### 4.2. Prediction Result’s Impact

Many research studies focus on achieving good surgery room usage time estimation but there are few discussions about considering a good or clinically acceptable performance. According to hospital management, efficiency leads to gaining high profit. Over estimation gives rise to more cost than under-estimation.

Clarke’s error grid (CEG) analysis is extensively used in blood glucose level estimation and acknowledged as the golden standard for evaluating model performance. We believe that Clarke’s error grid-like analysis method is useful for surgery room usage time estimation. CEG separates the scatter plot of prediction result versus reference value into different regions based on their implication [31]. The prediction result that falls in zone B does not lead to harmful decisions, and the false predictions in zone C, D, and E will lead to a potentially harmful outcome. In recent years, some studies have started to explore the impacts of prediction results. For example, Albert Wu et al. discussed the impact of overestimating surgical time on the medical system [32]. Based on the literature, the effect of different degrees of overestimations or underestimations, we have no doubt that there can also be CEG such as the golden standard for evaluating the OR room usage time estimation models performance based on the prediction’s impact.

### 4.3. Commonly Performed Procedures

From Figure 9c, we infer that the top five commonly performed procedures for poor-performing department 13 not only take longer procedures but also have larger variations when compared to other departments such as department 14. It is reasonable to assume that more complex procedures have more variables and take a long duration to complete the process which may require additional information to produce an accurate prediction result. This indicates that some departments with more complex procedures need more in-depth analysis of additional information that is in need to further improve the prediction performance on specific procedures. It seems that a more complex procedure would benefit from having its own model when sufficient data are available.

### 4.4. Practical Usage

In practical applications, the users would care about the schedule’s accuracy prediction and possible deviation in minutes. Hence, we suggest using the proportion of predicted results within a margin of minutes instead of the percentage of minutes for model evaluation. In this study, we demonstrate it with 5 and 10 minutes margin as shown in Table 3. From the table, we observe how each model performs in terms of the number of predictions, we expect it to be in the 5 or 10 minutes variation. Furthermore, we also presented the percentage of records that do not go overtime with the threshold of 5 (<5 min) and 10 (<10 min) minutes from the prediction result. With these two pieces of information, the user can more objectively assess the probability of surgery delay.

Our models only use features that are available before the surgery and avoid any ID-like features. Using these ID-like features such as doctor’s or nurse’s ID as a feature would significantly limit the universal usability of the model. The model needs to be retrained or modified when there is a new joining of an employee in certain unavoidable situations. Adding physiological information along with our features can increase the accuracy, however, this information may not always be available in the reality.

## 5. Conclusions and Future Work

In this study, we demonstrate different machine learning models that perform with only a little more than a dozen features that exclude any patient’s physiological information. In accordance with the previous literature, our XGBoost machine learning models outperform the other models that were tested for this study. This study finds the results of our best performing department-specific XGBoost model with the values 31.6 min, 18.71 min, 0.71, 28% and 27% for the metrics of root-mean-square error (RMSE), mean absolute error (MAE), coefficient of determination ($R^{2}$), mean absolute percentage error (MAPE) and proportion of estimated result within 10% variation, respectively. The aforementioned department-specific models also have 50% of their estimated time fall within 10 min variation from the actual value. However, there is 27% of the actual procedure time which exceeds the estimated time which is more than 10 min. While using fewer features for better universal usability, our model shows comparable performance with previous studies by using machine learning methods. We believe this result can bring improvements for OR room usage time estimation and benefit the current scheduling process positively.

Despite the neural-network-based models being outperformed by the decision-tree-based method, it does not show decision tree-based models are assured to be better. Our comparison between the two methods merely suggests that in this particular setting, the well fine-tuned decision tree algorithm surpasses the basic and most commonly used neural network structure. There are various different aspects of reasons and possibilities for the improvement of the neural network. One of the major difficulties of a neural-network-based model is high cardinality. Columns of procedure id has multiple unique values. Too many unique values in the categorical feature lead to high cardinality. We confer that with a more complex and well-designed architecture in combination with better organization and representation of the input data, the neural network can match or even outperform the decision tree models.

There are significant discrepancies in the performance between different department models. The discrepancies between them are affected by the time variation of the procedure. Other complex procedures have larger variations across a wide time frame and the lack of variables hinders determining the root cause. Further in-depth studies are required on the procedures with large variations to analyze the required information to improve estimation accuracy. It is reasonable to assume a more complex procedure would require more operation (OP) related features in order to produce accurate time estimation. We recommend building a procedure-specific model for each unique procedure with sufficient data to acquire the best possible performance. It will also help to determine the complex procedures that need more information to improve the prediction accuracy.

## Figures and Tables

**Table 1:** Data demographic.

Dept.	Sample Count	Doctors	Nurses	Unique OPs
16	21,968	58	182	367
29	20,476	52	169	266
14	14,763	52	185	602
17	13,566	72	179	652
22	13,338	60	178	674
24	12,410	69	185	279
11	9874	39	175	123
28	7670	46	168	448
13	4017	43	170	213
15	1965	8	155	141
12	1800	34	164	176
66	635	25	141	136
31	521	6	113	22
30	454	26	130	155
5	13	4	29	7
25	4	2	12	4
18	2	2	6	3
All	123,476	158	202	1916

**Table 2:** Results of all-inclusive models.

Method	RMSE	MAPE	MAE	$R^{2}$	±10%
	(min)	(%)	(min)		(%)
XGBoost	37.2	31	21.05	0.72	25
Random Forest	36.0	29	20.49	0.76	26
ANN	47.6	45	20.14	0.64	26
1-d CNN	48.6	47	27.57	0.63	19

**Table 3:** Department-Specific Model Result.

(a) Random Forest Model Results										
**Dept.**	**RMSE**	**MAE**	**MAPE**	$R^{2}$	±10% *	**±10 min** ${}^{*}$	±5 min *	<10 min **	<5 min **	**Sample_Count**
	**(min)**	**(min)**	**(%)**		**(%)**	**(%)**	**(%)**	**(%)**	**(%)**	
16	26.58	14.51	30	0.72	24	59	33	77	60	21,968
29	11.74	6.81	29	0.87	27	80	60	91	77	20,476
14	38.00	21.63	31	0.64	24	44	24	67	55	14,763
17	42.51	26.80	24	0.66	28	34	18	63	53	13,566
22	38.42	24.84	26	0.48	29	34	19	62	52	13,338
24	20.43	13.72	19	0.79	34	52	28	73	58	12,410
11	32.33	18.81	30	0.85	27	48	28	68	55	9874
28	47.86	25.88	31	0.68	26	40	22	66	56	7670
13	74.73	49.76	24	0.47	33	17	09	52	48	4017
15	35.63	15.11	21	−0.26	32	54	28	76	61	1965
12	62.21	43.15	28	0.67	24	22	10	53	45	1800
Average (weighted)	31.98	19.15	27	0.69	27	50	30	72	59	121,847
**(b) XGB Model Results**										
**Dept.**	**RMSE**	**MAE**	**MAPE**	$R^{2}$	±10% *	**±10 min** ${}^{*}$	±5 min *	<10 min **	<5 min **	**Sample_Count**
	**(min)**	**(min)**	**(%)**		**(%)**	**(%)**	**(%)**	**(%)**	**(%)**	
16	29.94	14.65	30	0.66	23	60	35	79	62	21,968
29	13.20	7.42	32	0.85	27	78	56	90	76	20,476
14	32.30	19.98	30	0.68	23	43	23	68	56	14,763
17	39.77	24.55	24	0.70	30	37	19	64	54	13,566
22	35.32	23.70	25	0.62	30	33	18	62	54	13,338
24	22.34	14.01	19	0.76	36	54	28	74	58	12,410
11	33.48	19.10	28	0.87	25	46	25	71	58	9874
28	51.31	28.14	33	0.71	22	36	18	64	54	7670
13	65.05	45.95	23	0.64	30	21	12	55	51	4017
15	35.50	15.55	22	−0.24	30	52	24	73	59	1965
12	56.67	38.27	26	0.73	31	25	09	56	47	1800
Average (weighted)	31.60	18.71	28	0.71	27	50	30	73	60	121,847

* proportion of samples within the ± variation; ** proportion of predicted samples within acceptable overtime in minutes.

**Table 4:** Comparison with previous studies.

Approach	RMSE	MAPE	MAE	$R^{2}$	±10%
	(min)	(%)	(min)		(%)
Linear Regression [18]	48.64	n/a	31.3	n/a	n/a
Ref. XGBoost [20]	n/a	27	n/a	0.77	32
Ref. Random Forest [20]	n/a	39	n/a	0.93	23
XGBoost-HY [21]	36.64	35.16	21.52	n/a	n/a
XGBoost-SH [21]	40.26	35.11	25.23	n/a	n/a
XGBoost - Department Specific (*w*)	31.60	28	18.71	0.71	27
Random Forest - Department Specific (*w*)	31.98	27	19.15	0.69	27
ANN - All inclusive	47.6	45	20.14	0.64	26
1-d CNN - All inclusive	48.6	47	28.77	0.65	17

**Figure A1:**
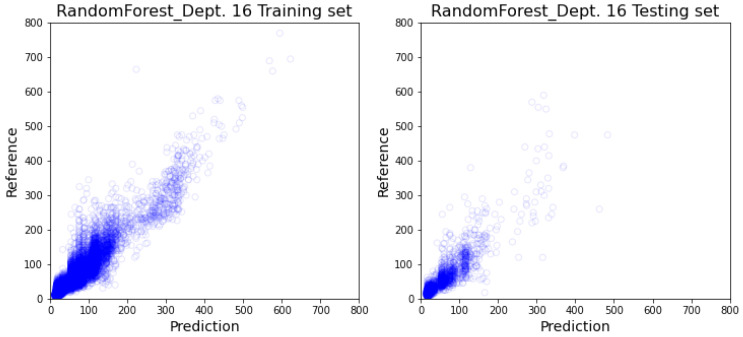
Model Performance of RandomForest on Dept.16. The visualization of training and testing data for department specific model on department 16 with Random Forest.

**Figure A2:**
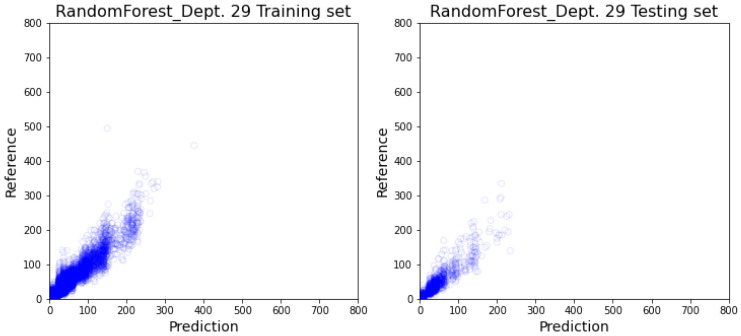
Model Performance of RandomForest on Dept.29. The visualization of training and testing data for department specific model on department 29 with Random Forest.

**Figure A3:**
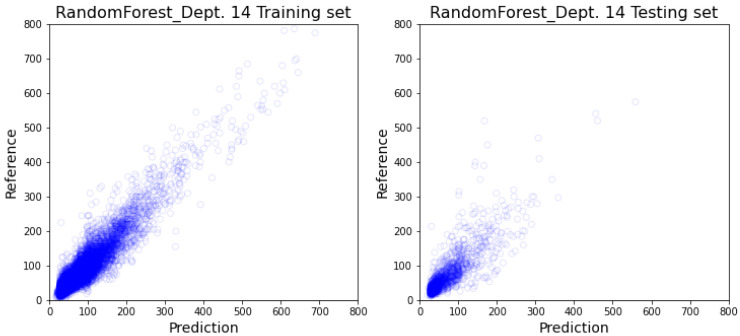
Model Performance of RandomForest on Dept.14. The visualization of training and testing data for department specific model on department 14 with Random Forest.

**Figure A4:**
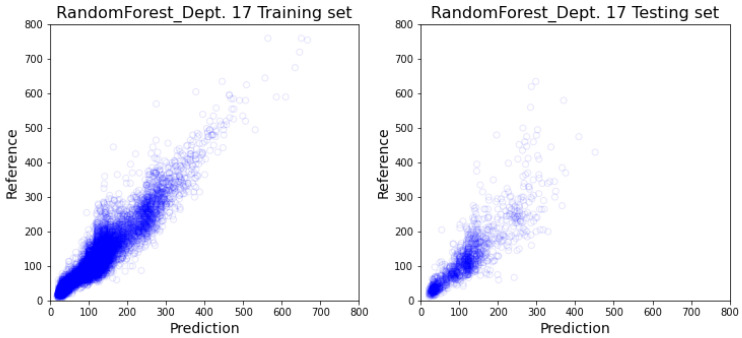
Model Performance of RandomForest on Dept.17. The visualization of training and testing data for department specific model on department 17 with Random Forest.

**Figure A5:**
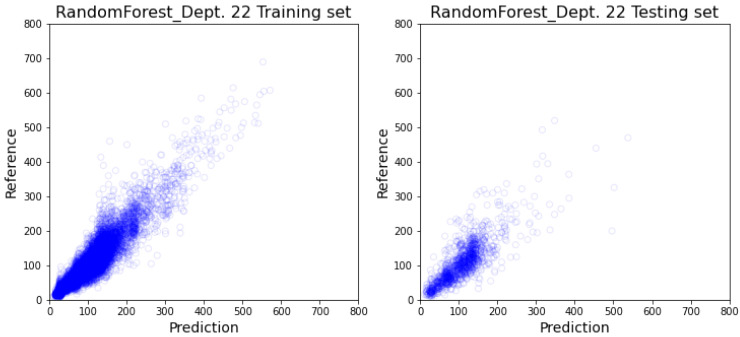
Model Performance of RandomForest on Dept.22. The visualization of training and testing data for department specific model on department 22 with Random Forest.

**Figure A6:**
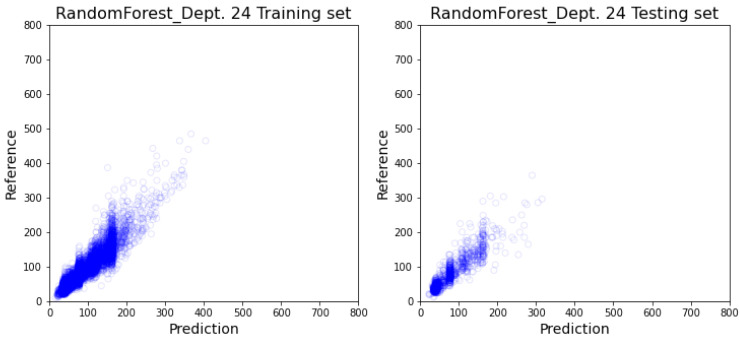
Model Performance of RandomForest on Dept.24. The visualization of training and testing data for department specific model on department 24 with Random Forest.

**Figure A7:**
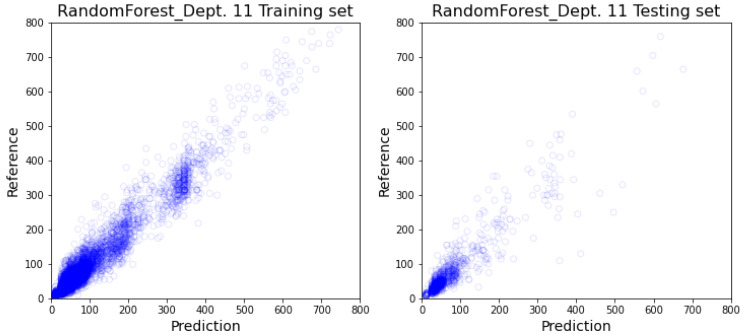
Model Performance of RandomForest on Dept.11. The visualization of training and testing data for department specific model on department 11 with Random Forest.

**Figure A8:**
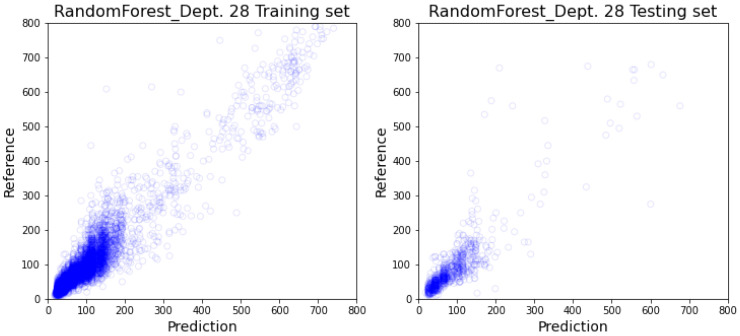
Model Performance of RandomForest on Dept.28. The visualization of training and testing data for department specific model on department 28 with Random Forest.

**Figure A9:**
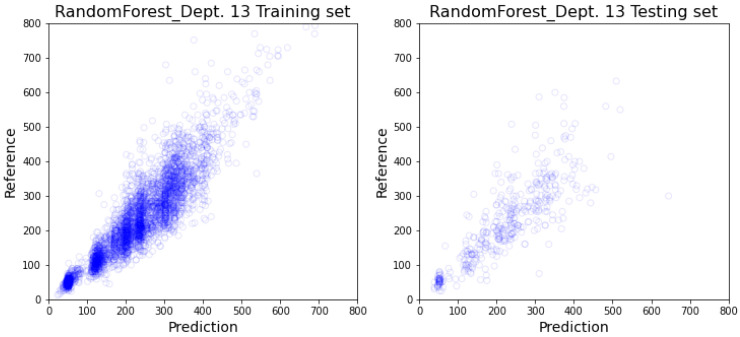
Model Performance of RandomForest on Dept.13. The visualization of training and testing data for department specific model on department 13 with Random Forest.

**Figure A10:**
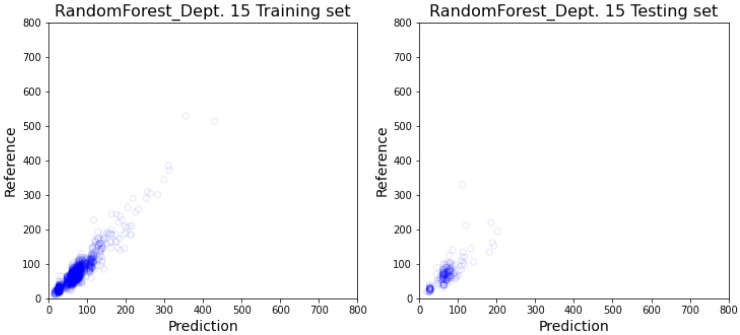
Model Performance of RandomForest on Dept.15. The visualization of training and testing data for department specific model on department 15 with Random Forest.

**Figure A11:**
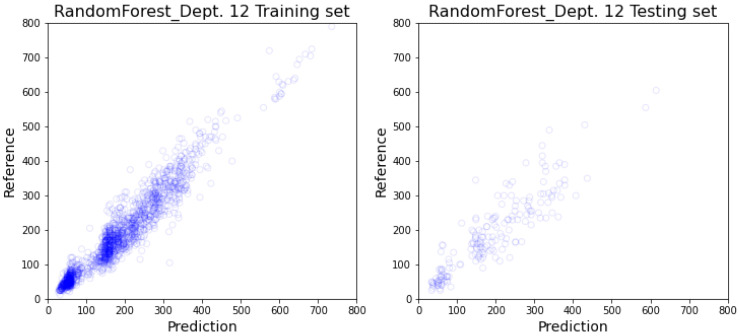
Model Performance of RandomForest on Dept.12. The visualization of training and testing data for department specific model on department 12 with Random Forest.

**Figure A12:**
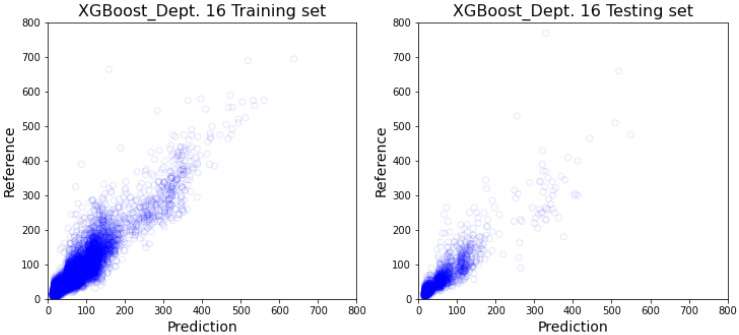
Model Performance of XGBoost on Dept.16. The visualization of training and testing data for department specific model on department 16 with XGBoost.

**Figure A13:**
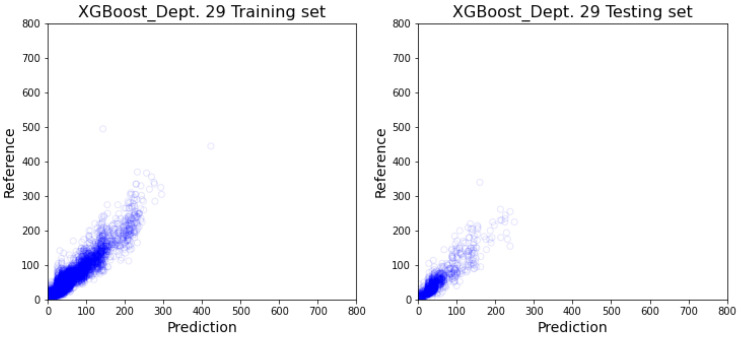
Model Performance of XGBoost on Dept.29. The visualization of training and testing data for department specific model on department 29 with XGBoost.

**Figure A14:**
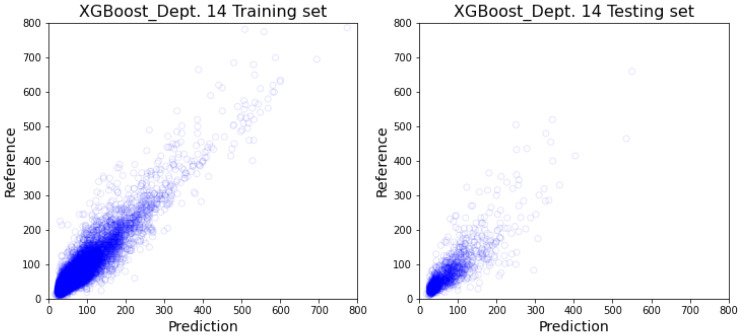
Model Performance of XGBoost on Dept.14. The visualization of training and testing data for department specific model on department 14 with XGBoost.

**Figure A15:**
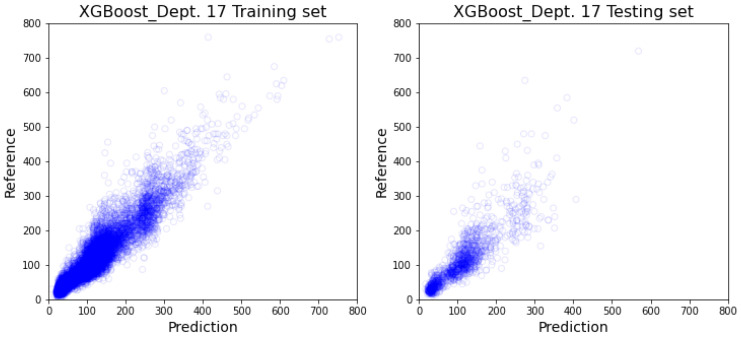
Model Performance of XGBoost on Dept.17. The visualization of training and testing data for department specific model on department 17 with XGBoost.

**Figure A16:**
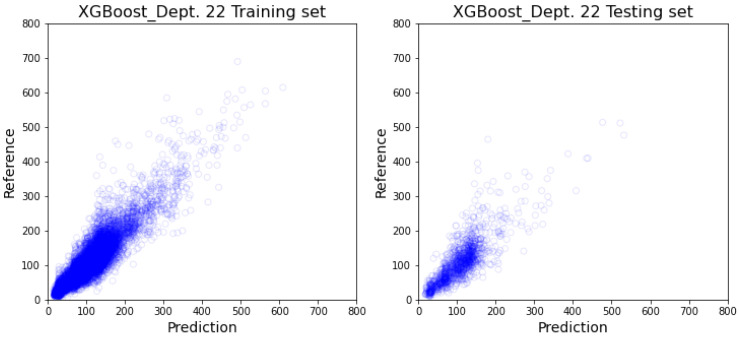
Model Performance of XGBoost on Dept.22. The visualization of training and testing data for department specific model on department 22 with XGBoost.

**Figure A17:**
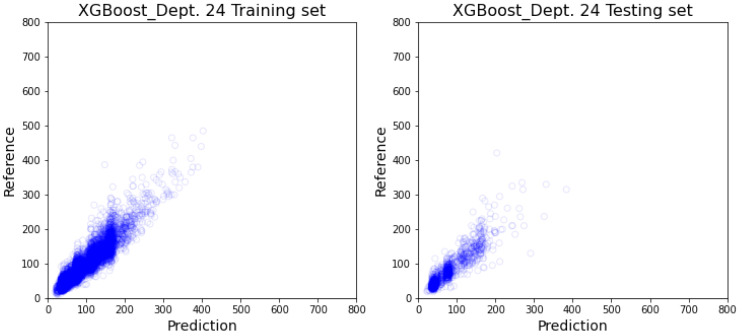
Model Performance of XGBoost on Dept.24. The visualization of training and testing data for department specific model on department 24 with XGBoost.

**Figure A18:**
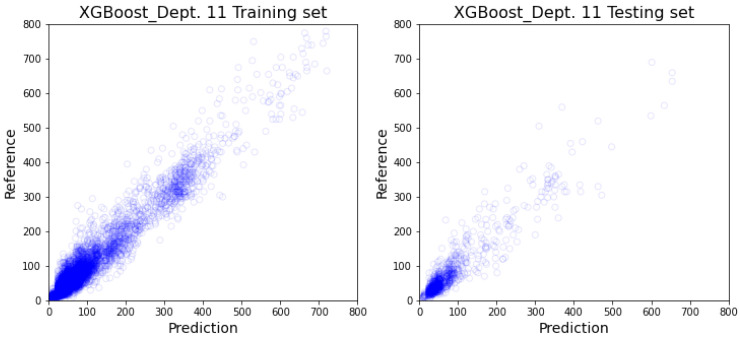
Model Performance of XGBoost on Dept.11. The visualization of training and testing data for department specific model on department 11 with XGBoost.

**Figure A19:**
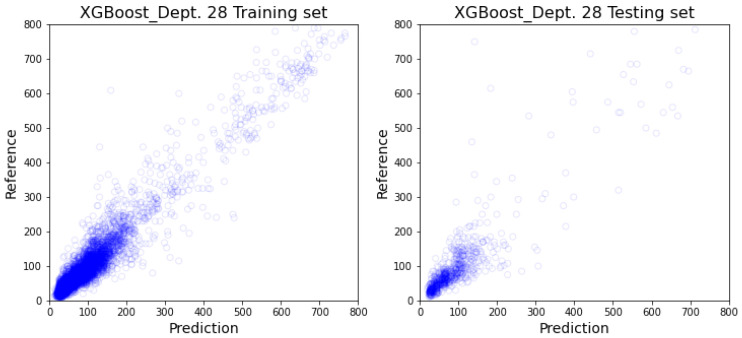
Model Performance of XGBoost on Dept.28. The visualization of training and testing data for department specific model on department 28 with XGBoost.

**Figure A20:**
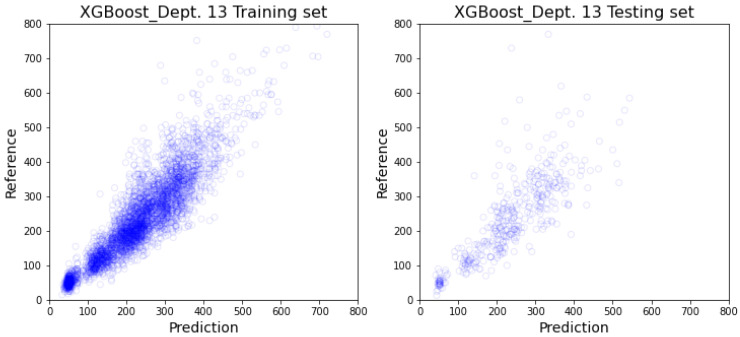
Model Performance of XGBoost on Dept.13. The visualization of training and testing data for department specific model on department 13 with XGBoost.

**Figure A21:**
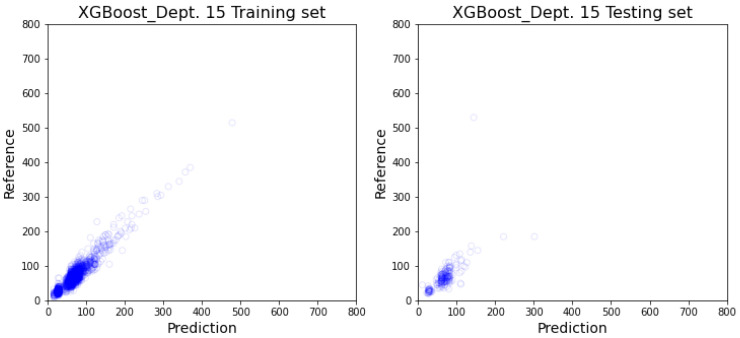
Model Performance of XGBoost on Dept.15. The visualization of training and testing data for department specific model on department 15 with XGBoost.

**Figure A22:**
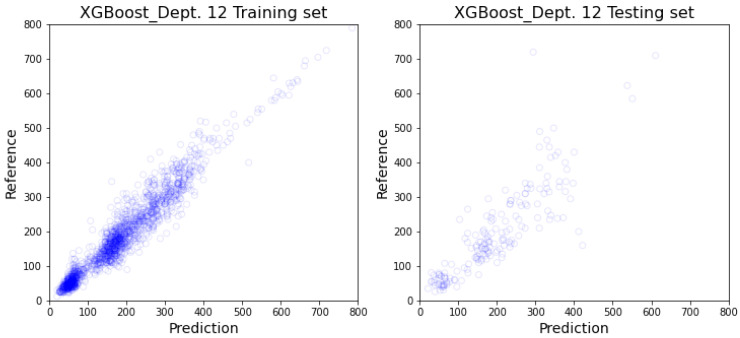
Model Performance of XGBoost on Dept.12. The visualization of training and testing data for department specific model on department 12 with XGBoost.

## Data Availability

The data generated during and analysed during the current study are available from the corresponding author on reasonable request.

## Appendix A

**Table A1:** Raw data feature columns with descriptions.

Column Namea	Descriptions
ODR_LOGN	Surgery serial number
ODR_CHRT	Chart Number
ODR_TXDT	Date
ODR_OPRM	Operating Room ID
ODR_DEPT	Department
ODR_PSRC	Patient Source (O: Outpatient, I: Inpatient, E: Emergency room)
ODR_BDNO	Bed number
ODR_EFLG	Emergency Surgery
ODR_IPNO	Inpatient Number
ODR_AS_D	Anesthesia Start (date)
ODR_AS_T	Anesthesia Start (time)
ODR_AE_D	Anesthesia End(date)
ODR_AE_T	Anesthesia End(time)
ODR_IN_D	Enter OR room (date)
ODR_IN_T	Enter OR room (time)
ODR_OS_D	Operation Start (date)
ODR_OS_T	Operation Start (time)
ODR_OE_D	Operation End (date)
ODR_OE_T	Operation End (time)
ODR_OT_D	Exit OR room (date)
ODR_OT_T	Exit OR room (time)
ODR_OP_1 ∼ODR_OP_4	Operating procedure type’s IDs
ODR_KF_2 ∼ODR_KF_4	N/A
ODR_SK_2 ∼ODR_SK_4	N/A
ODR_M_DR	Main Doctor
ODR_DN_1	scrub nurse 1
ODR_DN_2	scrub nurse 2
ODR_WN_1	circulation nurse 1
ODR_WN_2	circulation nurse 2
ODR_AD_1 ∼ODR_AD_4	N/A
ODR_PAYK	Payment category (01- Self-Pay, 30-Health insurance)
ODR_OPID	Operation ID
ODR_ANAM	Anesthesia methods
ODR_AN_D	Anesthesiologist (Doctor)
ODR_INDR	Anesthesia assessment Doctor
ODR_ASA	Anesthesia risk1∼5 (low∼high)
ODR_IRFG	N/A
ODR_IDFC	N/A
ODR_OPAG	N/A
ODR_FAID	N/A
ODR_DEAD	N/A
ODR_SAT1 ∼ODR_SAT5	N/A
ODR_ANS1 ∼ODR_ANS5	N/A
ODR_M_D2	Assistant Doctors 2 ∼4
ODR_PKN1 ∼ODR_PKN5	N/A
ODR_TIM1 ∼ODR_TIM5	N/A
ODR_CHRF	N/A
ODR_SPKD	N/A
ODR_ORMT	N/A
ODR_ANMT	N/A
ODR_ANT1 ∼ODR_ANT3	Anesthesia method’s notes
ODR_WOUD	Wound cleanness: 1: clean 2: cleaned contaminated 3: contaminated 4: dirty
ODR_ITE1 ∼ODR_ITE4	N/A
ODR_NPRO	N/A
ODR_PC01 ∼ODR_PC20	N/A
ODR_PRDG	Operating procedure name
ODR_FIND	Finding
ODR_OPF	Finding during procedure
ODR_OPP	Operating procedure
ODR_PODG	Post-operation diagnosis
